# The "Housing" Question

**Published:** 1902-01-25

**Authors:** 


### THE "HOUSING" QUESTION.

The subject of the housing of the working classes, which was selected by the Liberal member for North Camberwell as a suitable one upon which to found an amendment to the Address, is well calculated to lend itself to the oratory and the arguments of the party to which the honourable gentleman belongs. No one would be bold enough to deny that the homes of a large proportion of the population of the great towns, and of a considerable, although much smaller proportion of the populations of many country districts, are very far from attaining even the modest standard of healthiness and comfort which reasonable people might well desire to see universal, while it must at the same time be admitted that new and improved dwellings are seldom occupied by the class of persons who were displaced by clearing the ground for their construction, and who are not unfrequently forced to go farther afield, and to crowd together in slums as bad as, or possibly even worse than, those which they vacated. Such places become hotbeds of disease, and sources alike of moral and of physical contamination to all classes of the community. They are often centres of infection from which disease spreads through surrounding districts, and they furnish a perpetual stream of recruits to the ranks of prostitution and of professional criminality.

The difficulties in the way of better housing are mainly two, the first depending on the actual conditions of the building trade; the second on the peculiarities of the persons for whom improved homes are most urgently required. Under the influences—we will not say of trades-unionism, because that is an agency which, if it were wisely guided, might be of unmixed advantage to the community, but of the foolish principles by which much of the trades-unionism of the present day is guided and controlled—the cost of the labour required for the construction of a building is much greater than it ought to be, and constitutes an enormous tax upon an article of prime and universal necessity. Although ultimately, of course, this tax is paid by the tenant, it falls in the first instance upon the builder, who often strives to obtain compensation by the employment of the cheapest materials, and by winking at the worst forms of scamping in the way of workmanship. The "jerry-builder," in short, is the natural offspring of the trades-union "leader," and is in every way worthy of the stock from which he springs. Between them, dwellings for the working classes are "run up," wherever sites can be obtained for them, founded, not uncommonly, upon heaps of infected or fermenting rubbish, and constructed on the general principle of endeavouring to evade every provision of the Building Acts which cannot be defied. When a public body, such as a County Council, goes into the field, the prices alike of labour and of materials are at once raised against it, its surveyors refuse to pass materials and work which many private owners would accept, and the finished buildings are either too costly for the people for whom they are supposed to be intended, or else the losses incidental to low rents must be made good by the ratepayers of the district. The second difficulty is that the inhabitants of the cheapest dwellings cannot be prevented from destroying them. They will even tear gas-pipes and water-pipes out of the walls and sell them to obtain the price of gin. Such conditions are not to be altered by Acts of Parliament, but only by the gradual advance in education and civilisation of the people who are chiefly concerned. The remedies for them may, to some extent, be looked for in the school; and should be found there more and more as the teaching is more and more brought into harmony with Paley's definition of education, as comprising all the preparation which is made in youth for the sequel of our lives. Such preparation should at least be sufficient to preserve the average working man from becoming the dupe of political and socialistic agitators. Much may be done by employers, by the clergy, and by many of the various good influences which, in a variety of ways, are being brought to bear upon the lives of the poor by the labour and the self-devotion of some of their wealthier neighbours. Most of all could be done by the more intelligent of the working people themselves if they could but be brought to recognise the duty of striving to improve the standards of living among their own class. It is said that every nation is as well governed as it deserves to be; and although the individual must unfortunately suffer from his economic surroundings we are much inclined to think that speaking of whole nations or municipalities the principle which applies to governments will be found to apply equally to habitations.

